# Research on China insurance demand forecasting: Based on mixed frequency data model

**DOI:** 10.1371/journal.pone.0305523

**Published:** 2024-07-31

**Authors:** Cheng Wang, Mengnan Xu, Zheng Wang, Wenjing Sun

**Affiliations:** 1 School of Medical Economics and Management, Anhui University of Chinese Medicine, Hefei, China; 2 Key Laboratory of Data Science & Innovative Development of Traditional Chinese Medicine, Philosophy and Social Sciences of Anhui Province, Hefei, China; Royal Melbourne Institute of Technology, AUSTRALIA

## Abstract

In this paper, we introduce the mixed-frequency data model (MIDAS) to China’s insurance demand forecasting. We select the monthly indicators Consumer Confidence Index (CCI), China Economic Policy Uncertainty Index (EPU), Consumer Price Index (PPI), and quarterly indicator Depth of Insurance (TID) to construct a Mixed Data Sampling (MIDAS) regression model, which is used to study the impact and forecasting effect of CCI, EPU, and PPI on China’s insurance demand. To ensure forecasting accuracy, we investigate the forecasting effects of the MIDAS models with different weighting functions, forecasting windows, and a combination of forecasting methods, and use the selected optimal MIDAS models to forecast the short-term insurance demand in China. The experimental results show that the MIDAS model has good forecasting performance, especially in short-term forecasting. Rolling window and recursive identification prediction can improve the prediction accuracy, and the combination prediction makes the results more robust. Consumer confidence is the main factor influencing the demand for insurance during the COVID-19 period, and the demand for insurance is most sensitive to changes in consumer confidence. Shortly, China’s insurance demand is expected to return to the pre-COVID-19 level by 2023Q2, showing positive development. The findings of the study provide new ideas for China’s insurance policymaking.

## 1. Introduction

China’s insurance industry has maintained rapid growth for many years, and the scale, structure, and quality of insurance have been greatly improved, but compared with the developed countries in the world, China’s insurance industry is still at a relatively low level of development (see Table 1). As can be seen from Table 1, although the total premium income of China ranks the second level in the world, the insurance density and depth of insurance are far lower than that of developed countries, and even only half of the world’s average level, it can be said that the development potential of China’s insurance market is very large, and the demand for insurance is still strong, and the study of China’s insurance demand is of strong theoretical and practical significance.

**Table 1:** Comparison of insurance development levels in major countries worldwide in 2022.

Country (Region)	Premium Income (USD billion)	Share of global market (%)	Insurance density (USD)	Insurance depth (%)
USA	2960	43.6	8885	11.6
China	698	10.3	489	3.9
UK	363	5.4	4781	10.5
Japan	338	5.0	2690	8.2
France	261	3.9	3578	8.7
Germany	242	3.6	2881	5.9
Global	-	-	853	6.8

Source: Swiss Re, 2023, Sigma, No. 3.

Recent years have seen a global economic recession, rising unemployment and increasing uncertainty due to the impact of public health emergencies and systemic financial risk events. However, China has made excellent achievements in economic recovery, stabilizing financial markets, and maintaining regional stability. In this process, the insurance industry has given full play to the role of social “stabilizer” and economic “booster”, and the role of insurance in risk transfer, loss compensation, financial intermediation, and social security has become increasingly prominent. Therefore, studying the factors influencing the development of China’s insurance industry and accurately grasping the demand for insurance is crucial to ensuring the high-quality development of China’s economy.

In this paper, we attempt to construct a MIDAS model to explore the macro-influencing factors of China’s insurance demand, and at the same time make full use of mixed-frequency data information to forecast China’s insurance demand in real time. Multiple factors influence the demand for insurance, among which macroeconomic fluctuations risk, as a type of independent background risk, is considered an important factor affecting insurance purchase decisions [1]. The index of uncertainty in policies and economic conditions is an important indicator for measuring macroeconomic fluctuations in a country. The Economic Policy Uncertainty (EPU) index constructed by Baker et al. measures the degree of uncertainty in China’s economic policies [2]. Liu et al. and Ju et al. find that insurance demand increases with the fluctuation of economic policy uncertainty [3, 4]. At the same time, consumer expectations and actual disposable income levels also play an important role in affecting insurance premium expenditures [1, 5]. Therefore, we intend to use the Consumer Confidence Index (CCI), EPU, and Consumer Price Index (CPI) to construct a MIDAS model to predict the demand for insurance in China. Among them, CCI, EPU, and CPI are monthly high-frequency indicators, and insurance demand is measured using the quarterly low-frequency indicator of insurance depth (TID, regional premium income/regional GDP).

The contributions of this paper are threefold. First, the application of the MIDAS model in insurance demand forecasting enriches the research on the MIDAS model in the financial domain. Although there is relatively abundant research on the application of the MIDAS model in fields such as GDP forecasting and market volatility prediction [6–8], its application in the insurance domain remains comparatively limited. Furthermore, factors such as CCI, EPU, and CPI are generally considered to have an impact on insurance demand, although there is limited research on their predictive capacity concerning insurance demand. Second, Previous research on the MIDAS model focused mainly on its innovation and application. For instance, Kuzin et al. compared the prediction of Eurozone GDP using a mixed-frequency vector autoregressive model (MF-VAR) [9]. Aruoba et al. propose a mixed-frequency dynamic factor model (MF-DFM) that combines state-space models [10]. Foroni et al. propose reverse MIDAS (RR-MIDAS) and reverse U-MIDAS (RU-MIDAS) [11]. However, the performance of the model’s internal design and optimization has been neglected. We address this gap by considering the impact of different weight functions on the model and investigating the effects of three different prediction windows and different combination prediction forms on the prediction performance. Our findings lay a solid foundation for predicting future insurance demand. Thirdly, the optimal MIDAS model constructed in this paper is used for short-term forecasting and nowcasting of insurance demand in China. Previous studies mainly demonstrated the superiority and feasibility of different MIDAS models in the prediction field, without actually predicting the future.

The remainder of the paper is organized as follows. Section 2 Literature review. Section 3 The data and methodology. Section 4 Empirical results. Section 5 Nowcasting and short-term forecasting. Section 6 Conclusion and policy implications.

## 2. Literature review

### 2.1 Insurance demand influencing factors

Existing studies on insurance demand are mostly based on household microdata, examining the impact of factors such as household life cycle, financial status, asset mix, and gender on insurance demand. For example, Lin and Grace point out that there is significant heterogeneity in the demand for commercial life insurance among households in different life cycles, with older households allocating a lower proportion of commercial life insurance compared to younger households [12]; Tian and Dong point out that financial status directly affects the consumption demand for insurance, and different asset portfolios can create heterogeneity in the demand for insurance [13]; Wu and Zheng find that married female household heads are more inclined to purchase commercial insurance [14].

The above studies are based on the micro perspective, and macroeconomic volatility risk, as a separate contextual risk, has been identified as an important factor influencing insurance purchase decisions [1]. The index of uncertainty in policies and economic conditions is an important indicator for measuring macroeconomic fluctuations in a country. The Economic Policy Uncertainty (EPU) index constructed by Baker et al. measures the degree of uncertainty in China’s economic policies [2]. Liu et al. and Ju et al. find that insurance demand increases with the fluctuation of economic policy uncertainty [3, 4]. At the same time, consumer expectations and actual disposable income levels also play an important role in affecting insurance premium expenditures [1, 5]. Therefore, we intend to use the Consumer Confidence Index (CCI), EPU, and Consumer Price Index (CPI) to construct a MIDAS model to predict the demand for insurance in China. Among them, CCI, EPU, and CPI are monthly high-frequency indicators, and insurance demand is measured using the quarterly low-frequency indicator of insurance depth (TID, regional premium income/regional GDP).

### 2.2 MIDAS and forecasting

The prevailing prediction models are based on the co-frequency data, such as the autoregressive (AR) model, autoregressive moving average (ARMA) model, generalized autoregressive conditional heteroscedasticity (GARCH) model, back-propagation neural network model, and the grey model (GM) (1, N). While relying on the co-frequency model, prediction is often challenged by the synchronicity of data updates, leading to the need to convert indicator data frequencies. However, this conversion process: 1) leads to an information loss of high-frequency data, reducing the accuracy and timeliness of prediction; 2) may distort the structure of the low-frequency data, resulting in distortion of the constructed high-frequency data.

The Mixed Data Sampling (MIDAS) regression model proposed by Ghysels et al. effectively addresses the limitations of traditional prediction models, it incorporates variables of different frequencies into a single model by assigning weight functions to high-frequency data, to analyze the impact of high-frequency data on low-frequency data [15]. This method fully exploits the useful information contained in high-frequency data and enables advanced forecasting. Subsequently, the form and estimation methods of the mixed-frequency data sampling models have been continuously refined. Ghysels et al. introduced several mixed-frequency data sampling models, including the Multivariate Mixed Data Sampling model (M-MIDAS), Unrestricted Mixed Data Sampling model (U-MIDAS), Mixed Data Sampling model with Autoregressive terms (AR-MIDAS), and the Autoregressive Distributed Lag Mixed Data Sampling model (ADL-MIDAS) [16–19]. In the meantime, the MIDAS model has been widely applied in practical research. For example, Marcellino and Schumacher used dynamic and static estimation windows and the Factor-Augmented Mixed Data Sampling (FA-MIDAS) model to forecast Germany’s GDP [20]. Foroni et al. construct several MIDAS models and the U-MIDAS model using various weight functions to make real-time forecasts of US quarterly GDP [21]. Furthermore, the MIDAS model has also been used to predict inflation, energy consumption, price fluctuations, and others [22–27]. The existing literature demonstrates that the MIDAS model exhibits advantages not only in terms of prediction accuracy over its counterparts in frequency-based forecasting models, but also in its ability to achieve nowcasting and short-term forecasting, thus improving the timeliness of predictions [9, 28].

## 3. The date and methodology

### 3.1 Data processing

**Fig 1:**
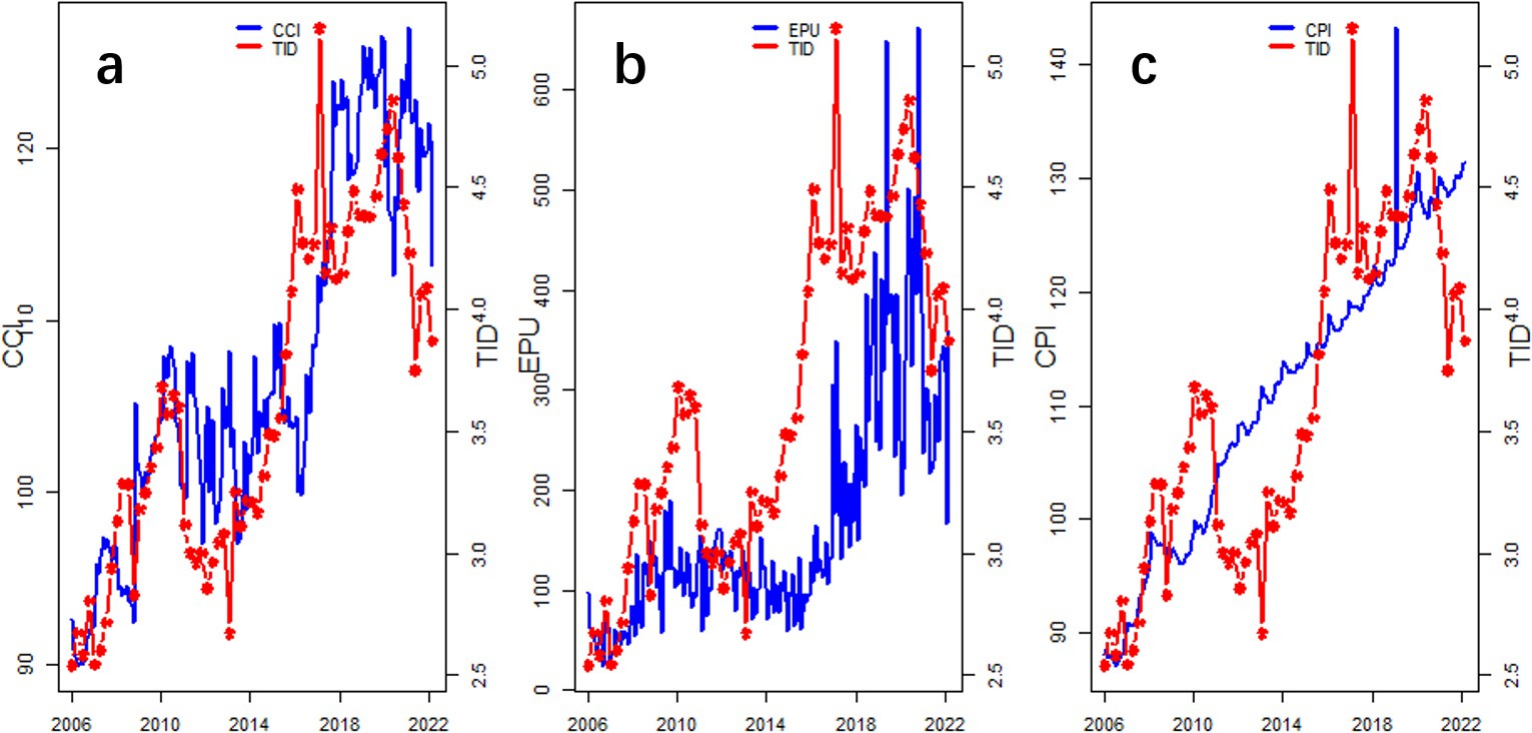
Row mixed frequency data. (a), (b) and (c) present the trends between the macroeconomic variables CCI, EPU, CPI and insurance demand TID, respectively, which basically show the same growth trends.

**Fig 2:**
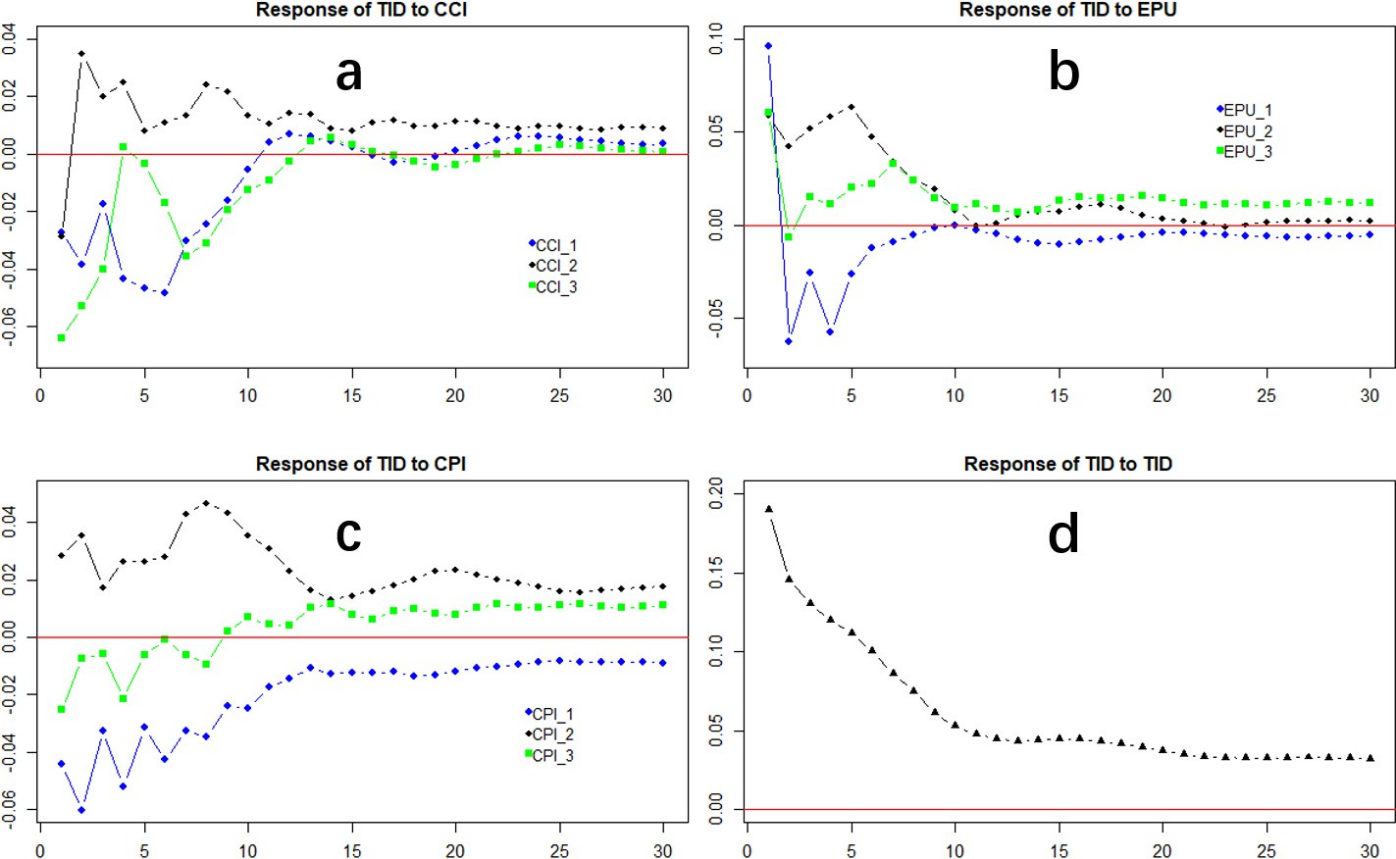
Response of TID to CCI, EPU& CPI. (a) Presents the impulse response of insurance demand TID to the macroeconomic variable CCI. (b) Presents the impulse response of insurance demand TID to the macroeconomic variable EPU. (c) Presents the impulse response of insurance demand TID to the macroeconomic variable CPI. (d) Presents the impulse response of insurance demand TID to the insurance demand TID.

The interval for the monthly indicators CCI, EPU, and CPI is 2006M1-2022M3, and the interval for the quarterly indicator TID is 2006Q1-2022Q1. where CCI and CPI are downloaded from the Wind database (www.wind.com.cn), and EPU is constructed using data from Baker et al. 2013, downloaded from www.policyuncertainty.com. To eliminate the seasonal periodicity of the time series data, we use the X-12-ARIMA method to seasonally adjust CCI, EPU, and CPI. Fig 1 shows the trend of TID with CCI, EPU, and CPI. Visually, both the explanatory and response variables exhibit a rising trend with oscillations, which suggests a certain correlation between TID and CCI, EPU, and CPI. Table 2 indicates that each explanatory variable is stable and can be applied directly to predict the MIDAS model. To further demonstrate the applicability of the selected explanatory variables, we conduct a mixed-frequency data impulse response experiment Fig 2 shows that the changes in CCI, EPU, and CPI have a significant impact on TID in the short term (generally within the first 10 periods), but maintain a stable relationship in the long term.

**Table 2:** Descriptive statistics.

Statistics	CCI	EPU	CPI
Observations	195	195	195
Mean	107.289	166.654	110.936
min	89.9	24.876	87.117
max	127	661.828	143.242
Std. dev.	10.268	116.596	12.857
skewness	0.339	1.571	-0.130
kurtosis	-1.007	2.525	-0.981
Jarque–Bera	0.003***	2.2e-16***	0.018**
ADF	0.009***	1.001e-10***	1.557e-09***

*** p<0.01

** p<0.05

* p<0.1

### 3.2 The methodology

#### 3.2.1 Model setup

The MIDAS model integrates the concept of distributed lag models (DL) and is consistent with the construction mechanism of bridging models. By applying a weighting function, high-frequency data can be incorporated into a low-frequency regression model, thus achieving the desirable outcome of coexisting data of varying frequencies.

We set the univariate *h*−*step* ahead prediction as follows:

$$y_{t}=\beta_{0}+\beta_{1}B(L^{1/m};\theta )x_{t-h}^{\left(m\right)}+\varepsilon_{t},$$ (1)

where *y*_*t*_ is the low-frequency explanatory variable in period *t*, and $x_{t}^{\left(m\right)}$ is the high-frequency explanatory variable, $B(L^{1/m};\theta )=\sum_{i=0}^{K}\omega (i;\theta )L^{i/m}$ is the weighted polynomial function, and $B(1;\theta )=\sum_{i=0}^{K}\omega (1;\theta )=1$, *L*^1/*m*^ is the lag operator for high-frequency data, $L^{i/m}x_{t-h}^{\left(m\right)}=x_{t-h/m-i/m}^{\left(m\right)},i=0,1,\cdots K-1$, *ε*_*t*_ is a random disturbance term, *m* denotes the frequency ratio between the high-frequency variable and the low-frequency variable, in this paper we set *m* = 3. *K* is the lag order of high-frequency data, we set the highest lag order *K*−1 to 30. *h* is the prediction horizon, it represents the mixed frequency data sampling of one step forward when *h* = 1, that is, the high-frequency data of the first two months of the current quarter are used to predict the low-frequency data of the current quarter; when *h*>3, out-of-sample prediction can be realized.

We construct the MIDAS model using high-frequency explanatory variables CCI, EPU, and CPI and explain variable TID and the lag effect of TID is considered. The following expression can be obtained:

$$TID_{t}=\beta_{0}+\beta_{1}B_{1}(L^{1/3};\theta )CCI_{t-h}^{\left(3\right)}+\beta_{2}B_{2}(L^{1/3};\theta )EPU_{t-h}^{\left(3\right)}+\beta_{3}B_{3}(L^{1/3};\theta )CPI_{t-h}^{\left(3\right)}+\beta_{4}TID_{t-p}+\varepsilon_{t},$$ (2)

when there is no weight function, it is the U-MIDAS model, which can be expressed as:

$$\begin{array}{l} TID_{t}=\beta_{0}+\beta_{1,0}CCI_{t}+\beta_{1,1}CCI_{t-1/3}+\beta_{1,2}CCI_{t-2/3}+\beta_{1,3}CCI_{t-1}+\cdots +\beta_{1,3\times q_{1}}CCI_{t-q_{1}}\\ \;+\beta_{2,0}EPU_{t}+\beta_{2,1}EPU_{t-1/3}+\beta_{2,2}EPU_{t-2/3}+\beta_{2,3}EPU_{t-1}+\cdots +\beta_{1,3\times q_{2}}EPU_{t-q_{2}}\\ \;+\beta_{3,0}CPI_{t}+\beta_{3,1}CPI_{t-1/3}+\beta_{3,2}CPI_{t-2/3}+\beta_{3,3}CPI_{t-1}+\cdots +\beta_{1,3\times q_{3}}CPI_{t-q_{3}}\\ \;+\beta_{4}TID_{t-p}+\varepsilon_{t} \end{array},$$ (3)

where *q*_1_, *q*_2_, *q*_3_ is the lag order of high-frequency data, and is a random disturbance term.

#### 3.2.2 Weighting function

The key to the MIDAS data structure is frequency alignment, it is sufficient to treat each high-frequency variable $x_{t-h}^{\left(m\right)}$ observation corresponding to a low-frequency variable *y*_*t*_ as the same. It is easy to achieve for a small frequency ratio but will be cumbersome for a larger frequency ratio. e.g., if *y*_*t*_ is monthly data and $x_{t-h}^{\left(m\right)}$ is daily data, then for each *y*_*t*_, there will be at least 21 observations $x_{t-h}^{\left(m\right)}$ corresponding to it. In this case, it is necessary to introduce a polynomial weight function *ω*(*k*;*θ*) to constrain the high-frequency variables, and the optimal effect of the MIDAS model can be achieved through the selection of parameter vector *θ* and lag order *K*. In other words, the information of high-frequency data can be retained and the number of parameters to be estimated can be reduced. In this way, the high-frequency data information can be retained, the number of parameters to be estimated can be reduced, and the data-driven automatic screening of the appropriate maximum lag order *K* avoids the problem of lag order selection in the case of no parameter constraints. Ghysels et al. find that the Beta weight function, Almon weight function, and Exp Almon weight function are more frequently used and generally have better prediction results [18]. In this paper, we examine the predicted results of each weighting function. The weighting function can be expressed as follows:

Beta weight function

$$\omega (k;\theta_{1},\theta_{2})=\frac{f(k/K;\theta_{1},\theta_{2})}{\sum_{k=1}^{K}f(k/K;\theta_{1},\theta_{2})},$$ (4)

where

$$f(X;a,b)=\frac{X^{a-1}{\left(1-X\right)}^{b-1}\Gamma (a+b)}{\Gamma (a)\Gamma (b)},\Gamma (a)=\int_{0}^{\infty }e^{-x}x^{a-1}dx.$$ (5)

The Beta polynomial weight function is derived from the probability density function in the family of Beta distributions [29], The different values of *θ*_1_,*θ*_2_ enable the generation of incremental, decreasing, and then increasing forms of weight changes. Beta weighting functions are used more often in the prediction and analysis of financial market volatility [8].

Almon weight function

$$\omega (k;\theta )=\theta_{0}+\theta_{1}k+\theta_{2}k^{2}+\cdots +\theta_{p}k^{p}.$$ (6)

Exp Almon weight function

$$\omega (k;\theta )=\frac{\exp(\theta_{0}+\theta_{1}k+\theta_{2}k^{2}+\cdots +\theta_{p}k^{p})}{\sum_{k=1}^{K}\exp(\theta_{0}+\theta_{1}k+\theta_{2}k^{2}+\cdots +\theta_{p}k^{p})}.$$ (7)

Almon weight function and Exp Almon weights are some of the most commonly used polynomial functional forms that allow the construction of a wide range of weight functions with a guaranteed positive number of weights, which gives the equations the nice property of having zero approximation error [17] Two parameter exponential Almon lagging polynomials are often used in macroeconomic studies. The *θ*_1_≤300, *θ*_2_<0 are generally carried out to satisfy the macroeconomic analyses and macroeconomic forecasting required by the weight forms [28].

#### 3.2.3 Combination forecasting and weighting criteria

We know that combined forecasting generally improves the accuracy and reliability of forecasting relative to single indicator forecasting, so we also investigated the estimation effects of univariate MIDAS models in different combined forms. The expression for combination forecasting, derived from multiple univariate predictors, is as follows:

$${\hat{f}}_{t+h|t}=\sum_{j=1}^{n}\omega_{j,t}{{\hat{y}}^{A}}_{j,t+h|t},$$ (8)

where *n* represents the total number of explanatory variables based on univariate predictions, ${{\hat{y}}^{A}}_{j,t+h|t}$ denotes the value of the forward *h*−*step* prediction in period *t*, *ω*_*j*,*t*_ represents the weight of the combined prediction function, and the usual methods for determining the weights include the following:

EW weighting method:

$$\omega_{j,t}=1/n.$$ (9)

BIC weighting method:

$$\omega_{j,t}=\frac{\exp(-BIC_{j})}{\sum_{j=1}^{n}\exp(-BIC_{j})}.$$ (10)

MSFE and DMSFE weighting method:

$$\omega_{j,t}=\frac{m_{j,t}^{-1}}{\sum_{j=1}^{n}m_{j,t}^{-1}},m_{j,t}=\sum_{j=T_{0}}^{t}\delta^{t-j}{\left(y_{s+h}^{h}-{\hat{y}}_{j,s+h|s}^{h}\right)}^{2},$$ (11)

where *T*_0_ corresponds to the starting point of the in-sample prediction. When *δ* = 1, it corresponds to the MSFE weight, and when *δ* = 0.9, it is the DMSFE weight.

#### 3.2.4 Evaluation criteria

The prediction accuracy is measured by the root mean square error (RMSE). The expression of RMSE is:

$$RMSE=\sqrt{\frac{\sum_{t=1}^{T}{\left(Y_{t}-{\hat{Y}}_{t}\right)}^{2}}{T}}.$$ (12)

In general, the smaller the values of RMSE, the better performance of the model.

## 4. Empirical results

### 4.1 MIDAS model selection

The selection of the weighting function and its optimal lag order is crucial for the MIDAS model. We construct three MIDAS models using the weighting above functions, in addition to the U-MIDAS model, resulting in four models in total. The selection criteria for the weighting function are as follows: firstly, a mixed-frequency data regression model is constructed for the insurance demand variable TID and a single explanatory variable $x_{t-h}^{\left(m\right)}$, to obtain the optimal lag order of the model’s weighting function for the high-frequency data, in which the lag order for the high-frequency variables is set from 1 to 30. The optimal model is determined based on the Akaike information criterion (AIC). Considering the possible autocorrelation of the explanatory variable TID, we included TID(-1) in the regression model. Secondly, considering that the COVID-19 event may have an impact on the model fitting effect, for this reason, we exclude the COVID-19 (from 2019Q3 to 2022Q1) period data for re-estimation. Finally, the COVID-19 period sample is selected to test the forecasting effect of the selected optimal model.

**Fig 3:**
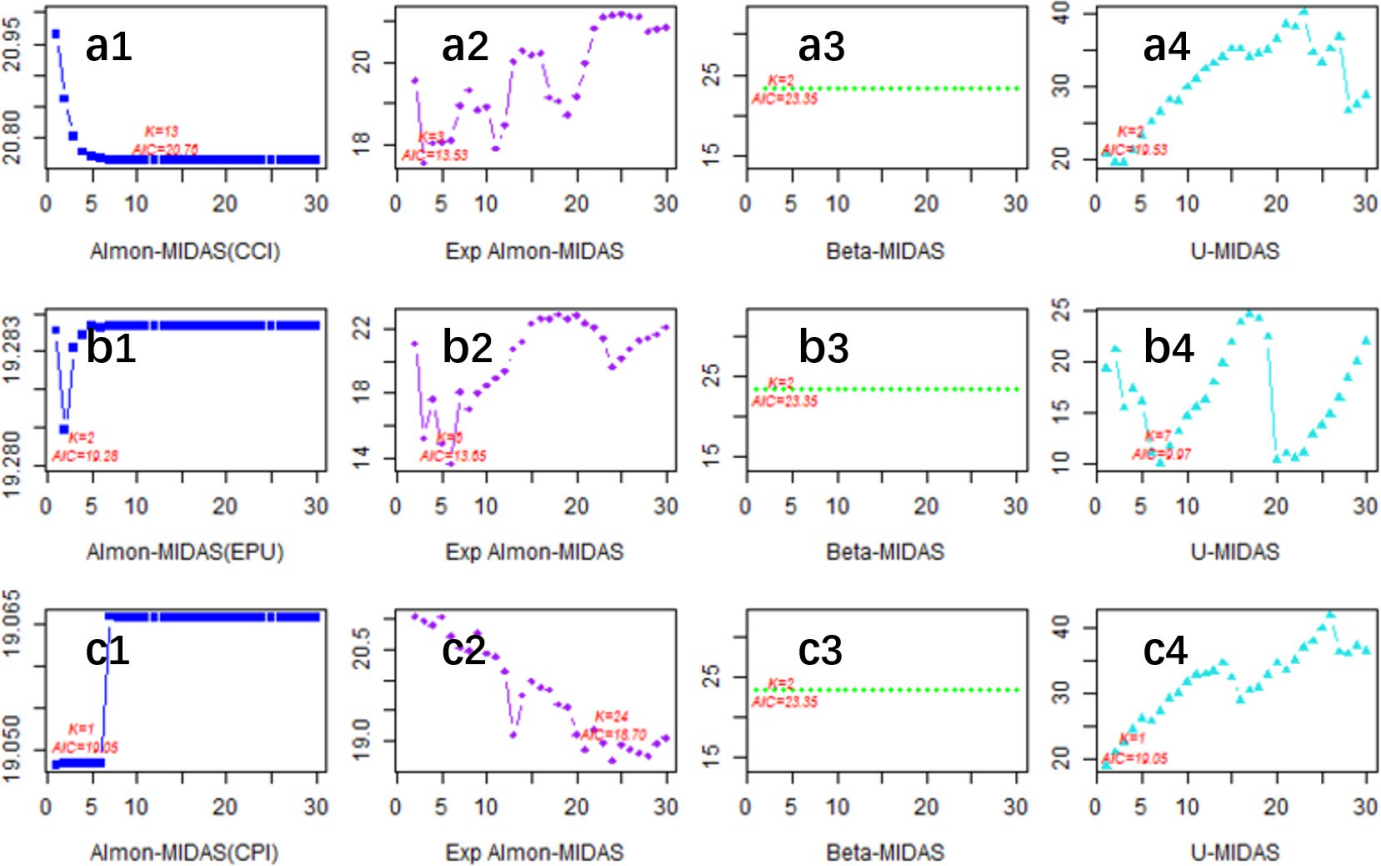
Optimal model selection (full sample). (a1)-(a4) present the AIC values corresponding to the 1–30 period lag order of the macroeconomic variable CCI in the Almon-MIDAS model, ExpAlmon-MIDAS model, Beta-MIDAS model, and U-MIDAS model. (b1)-(b4) present the AIC values corresponding to the 1–30 period lag order of the macroeconomic variable EPU for the Almon-MIDAS model, ExpAlmon-MIDAS model, Beta-MIDAS model, and U-MIDAS model. (c1)-(c4) present the AIC values corresponding to the 1–30 period lag order of the macroeconomic variable CPI in the Almon-MIDAS model, ExpAlmon-MIDAS model, Beta-MIDAS model, and U-MIDAS model.

Fig 3 represents the optimal univariate MIDAS model based on the full sample. The optimal MIDAS models for the high-frequency explanatory variables CCI, EPU, and CPI are Exp almon-AR(1)-MIDAS(3), U-AR(1)-MIDAS(7), and Exp almon-AR(1)-MIDAS(24), respectively, which indicate that the optimal lag orders in the full-sample estimation are 3, 7, and 24 months for CCI, EPU, and CPI, respectively.

**Fig 4:**
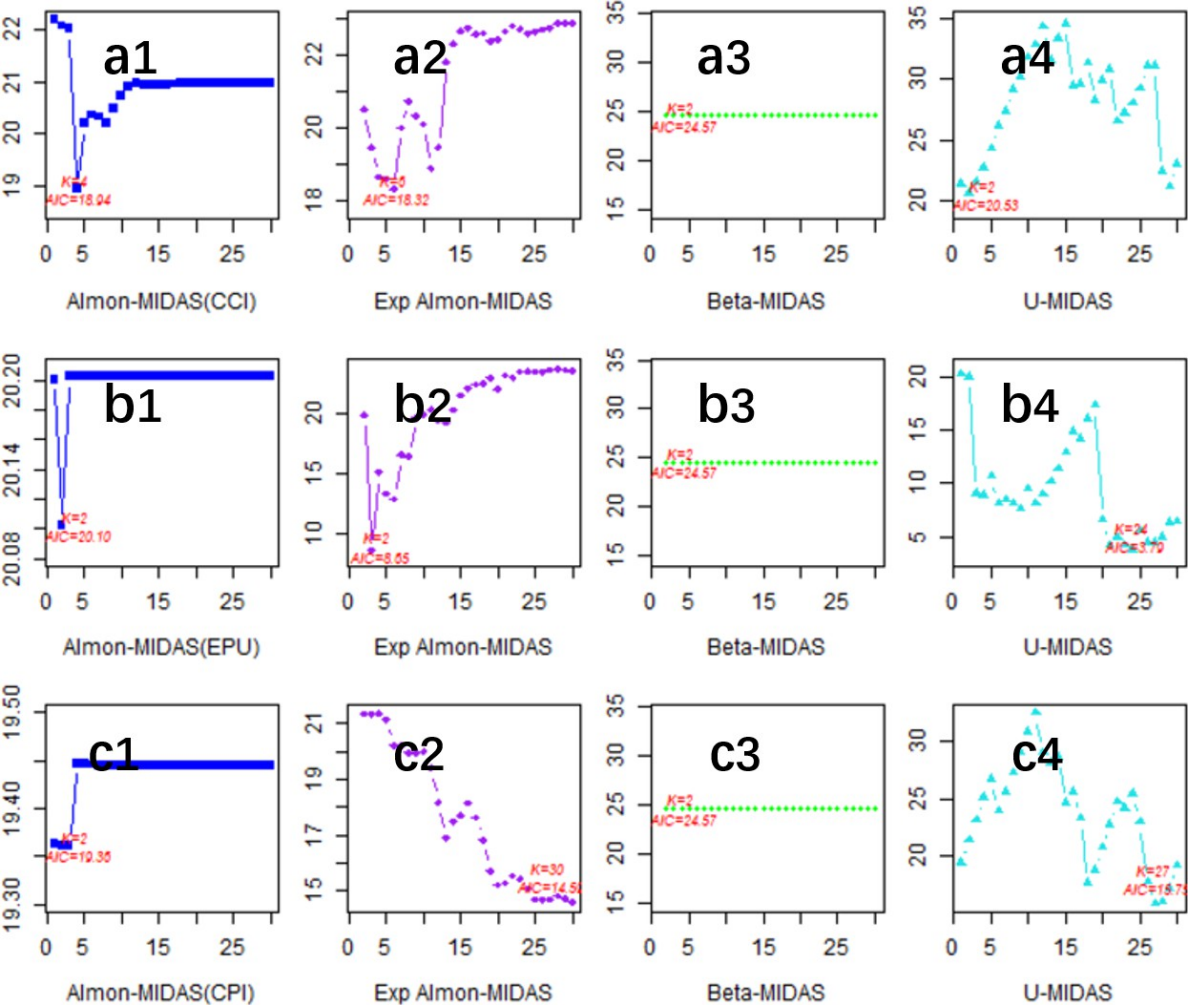
Optimal model selection (in-sample). (a1)-(a4) present the AIC values corresponding to the 1–30 period lag order of the macroeconomic variable CCI in the Almon-MIDAS model, ExpAlmon-MIDAS model, Beta-MIDAS model, and U-MIDAS model. (b1)-(b4) present the AIC values corresponding to the 1–30 period lag order of the macroeconomic variable EPU for the Almon-MIDAS model, ExpAlmon-MIDAS model, Beta-MIDAS model, and U-MIDAS model. (c1)-(c4) present the AIC values corresponding to the 1–30 period lag order of the macroeconomic variable CPI in the Almon-MIDAS model, ExpAlmon-MIDAS model, Beta-MIDAS model, and U-MIDAS model.

Fig 4 represents the effect of fitting based on in-sample data. The optimal MIDAS models corresponding to the high-frequency explanatory variables CCI, EPU, and CPI in the in-sample estimation are Exp almon-AR(1)-MIDAS(6), U-AR(1)-MIDAS(24), and Exp almon-AR(1)-MIDAS(30), respectively, which indicate that the optimal lags of CCI, EPU, and CPI in the in-sample estimation are 6, 24, and 30 months, respectively. The optimal lags for the CCI, EPU, and CPI in the in-sample estimates are 6, 24, and 30 months, respectively. It is worth noting that the change in sample period does not change the type of optimal weight function, but increases the optimal lag order of each high-frequency explanatory variable, suggesting that the TID has become more sensitive to the effects of CCI, EPU, and CPI as a result of the COVID-19 crisis. In addition, these results suggest that the Exp Almon-MIDAS model and the U-MIDAS model outperform the other models.

### 4.2 Benchmark model comparison

In this section, we divide the whole sample period into the sample estimation period (from 2006Q1 to 2019Q2) and the short-term forecasting period (from 2019Q3 to 2022Q1), and analyze the forecasting performance of the selected optimal univariate MIDAS model and multivariate MIDAS model in the COVID-19 period in comparison with the traditional AR and ARDL models of the co-frequency. Table 3 presents the root mean square error values of the in-sample predictions of the TID explanatory variables under the optimal weighting function and its optimal lag order. The results show that the MIDAS model outperforms the co-frequency AR model and the ARDL model in terms of in-sample prediction accuracy. In addition, the MIDAS model outperforms the same-frequency model in terms of in-sample prediction, except for the univariate EPU. It is worth noting that the in-sample prediction accuracy is lower than the in-sample estimation accuracy due to the effect of COVID-19, especially for EPU, whose optimal lag order for in-sample prediction is shorter, which indicates that during COVID-19, the change of EPU has a relatively small effect on the TID, and the TID is less sensitive to the change of EPU. (As shown in Table 3, when the EPU indicator is excluded, the M(2)-AR(1)-MIDAS model is better than the covariate AR model and ARDL model, except for the univariate EPU. AR(1)-MIDAS function is better when the EPU indicator is excluded, as shown in Table 3). On the contrary, the in-sample prediction accuracy of CCI is better than the in-sample estimation accuracy, indicating that CCI is the main factor affecting the change of TID during COVID-19, and TID is more sensitive to the change of CCI.

**Table 3:** Optimal in-sample prediction results.

Variables	Models	RMSE
CCI	AR(1)-MIDAS(3,6)	0.2447
EPU	AR(1)-MIDAS(3,24)	0.8645
CPI	AR(1)-MIDAS(3,30)	0.3085
M(3)	M(3)-AR(1)-MIDAS	0.2759
M(2)	M(2)-AR(1)-MIDAS	0.2375
-	AR(2)	0.4811
M(3)	ARDL(1,0,0,0)	0.3608

M(2) represents the model consisting of the explanatory variables CCI, CPI

M(3) represents the model consisting of the explanatory variables CCI, EPU, and CPI.

**Fig 5:**
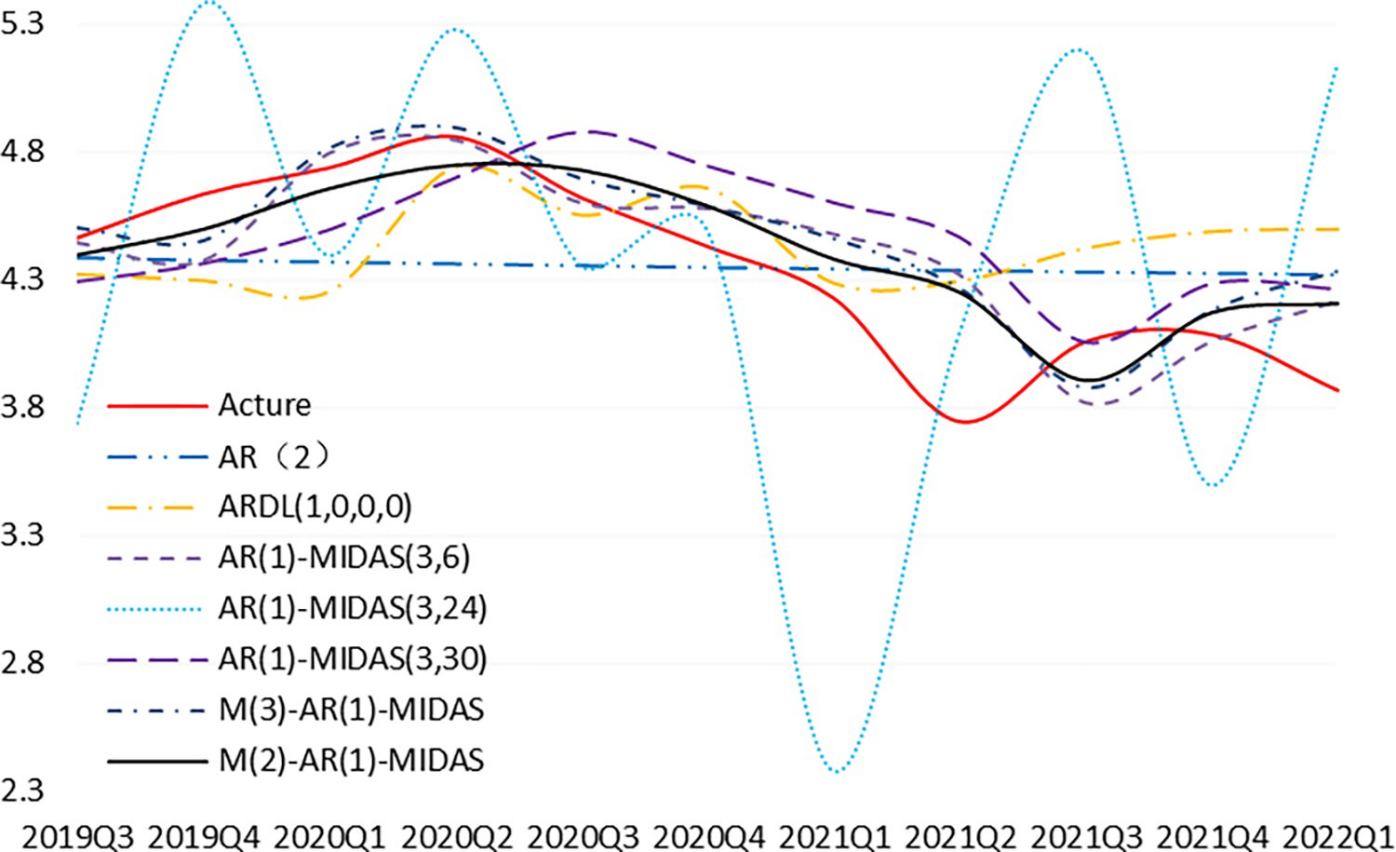
Comparison of in-sample predictions. The figure presents the optimal co-frequency model, mixed-frequency model (univariate, multivariate) based forecasts compared to the true values for the period 2019Q3-2022Q1. Following the legend from top to bottom, the true values, AR model forecasts, ARDL model (CCI, EPU and CPI) forecasts, Eep Almon-MIDAS (CCI) model forecasts, U-MIDAS (EPU) model forecasts, Eep Almon-MIDAS (CPI) model forecasts, and M-MIDAS (CCI, EPU and CPI) model forecasts, and M-MIDAS (CCI and CPI) model forecasts. Of course these models are based on the results under the optimal lag order and optimal weight function. From the graphs, it is obvious that the MIDAS model predictions are generally better than the co-frequency model predictions. 4.3 Optimization of MIDAS estimation methods.

Fig 5 shows the in-sample predictions of the co-frequency model and the MIDAS model. It is clear from Fig 5 that the MIDAS model outperforms the co-frequency AR model and the ARDL model in terms of prediction. In addition, the overall performance of the multivariate MIDAS model is better than that of the univariate MIDAS model, while the M(2)-AR(1)-MIDAS model without the EPU variable has the best fitting performance.

For the same MIDAS model, different prediction windows can also affect the prediction performance. Based on the three optimal models selected above, we examined the sample in-sample prediction performance under three prediction windows: fixed, rolling, and recursive. Table 4 presents the RMSE values for the sample in-sample prediction of 1 to 9 periods ahead based on CCI, EPU, and CPI under different prediction windows. From Table 4, we can see that the overall prediction performance of the MIDAS model is good, with RMSE values kept below 1.6118, and the lowest value is 0.2226. Among them, the prediction performance of the CCI indicator is the best, with the smallest and most stable RMSE value. The prediction performance of the EPU indicator is the worst, with a relatively high RMSE value. At the same time, the accuracy of the prediction gradually decreases with increasing prediction period *h*, which indicates that the MIDAS model is more suitable for short-term prediction. Also, different prediction windows have different effects on prediction performance. Generally speaking, the prediction accuracy of the rolling window and recursive window is better than the accuracy fixed window, but there are differences for single-variable prediction. For CCI, the rolling window prediction performance is better in the short term, the fixed window prediction performance is better in the medium term, and the recursive identification is better in the long term. For the EPU, the fixed window prediction performance is better in the short term, and the recursive window performs better in the medium to long term. For the CPI, the prediction performance of the recursive window is the best.

**Table 4:** RMSE values of in-sample predictions under different prediction windows.

horizon	CCI			EPU			CPI		
	fixed	rolling	recursive	fixed	rolling	recursive	fixed	rolling	recursive
h = 1	0.2264	0.2226	0.2250	0.8435	0.9267	0.8469	0.3315	0.3661	0.3068
h = 2	0.2315	0.2279	0.2314	0.8464	0.9832	0.8369	0.3288	0.3658	0.3063
h = 3	0.2282	0.2306	0.2320	1.1485	1.1926	1.0206	0.3625	0.3587	0.3241
h = 4	0.2313	0.2343	0.2350	1.0304	1.0607	0.9705	0.3528	0.3624	0.3187
h = 5	0.2284	0.2364	0.2361	1.0402	1.1279	0.9611	0.3548	0.3629	0.3219
h = 6	0.2320	0.2441	0.2408	1.2505	1.3311	1.0941	0.3356	0.3551	0.2987
h = 7	0.2415	0.2540	0.2487	1.2445	1.2471	1.0183	0.3163	0.3936	0.2929
h = 8	0.2560	0.2658	0.2562	1.3769	1.2105	0.9813	0.3042	0.3885	0.2854
h = 9	0.2832	0.2742	0.2670	1.6118	1.3729	1.0980	0.3426	0.3945	0.3072

The black font indicates the optimal forecast level for the same period.

Table 5 shows the RMSE values for the four combined forms of EW, BIC, MSFE, and DMSFE for periods 1 to 9. Based on the results presented in Table 5, it can be observed that the combined forecasting approach performs favorably, as evidenced by the stable and reasonable range of RMSE values, which are generally low. Specifically, the BIC-weighted approach appears to yield the most accurate forecasts, and when applied in conjunction with a short-term rolling window, it exhibits superior performance compared to long-term recursive prediction.

**Table 5:** RMSE values for combined forecasting.

horizon	Scheme	EW	BIC	MSFE	DMSFE
h = 1	fixed	0.3296	0.2159	0.2367	0.2402
	rolling	0.2668	0.2019	0.2263	0.2296
	recursive	0.2513	0.2030	0.2203	0.2227
h = 2	fixed	0.3441	0.2596	0.2711	0.2733
	rolling	0.2889	0.2419	0.2649	0.2675
	recursive	0.2709	0.2421	0.2540	0.2563
h = 3	fixed	0.3506	0.2618	0.2804	0.2820
	rolling	0.2976	0.2587	0.2756	0.2773
	recursive	0.2785	0.2582	0.2625	0.2641
h = 4	fixed	0.2788	0.2654	0.2662	0.2661
	rolling	0.2746	0.2703	0.2641	0.2626
	recursive	0.2496	0.2627	0.2494	0.2485
h = 5	fixed	0.2760	0.2327	0.2468	0.2481
	rolling	0.2553	0.2421	0.2375	0.2367
	recursive	0.2296	0.2345	0.2268	0.2262
h = 6	fixed	0.2972	0.2335	0.2576	0.2597
	rolling	0.2742	0.2353	0.2491	0.2498
	recursive	0.2497	0.2333	0.2392	0.2400
h = 7	fixed	0.2901	0.2199	0.2444	0.2437
	rolling	0.2791	0.2315	0.2515	0.2524
	recursive	0.2638	0.2303	0.2465	0.2480
h = 8	fixed	0.2739	0.2371	0.2436	0.2429
	rolling	0.2959	0.2357	0.2650	0.2663
	recursive	0.2863	0.2349	0.2617	0.2632
h = 9	fixed	0.4992	0.3417	0.2806	0.2808
	rolling	0.4477	0.3931	0.2854	0.2830
	recursive	0.3962	0.3067	0.2712	0.2705

The black font indicates the optimal forecast level for the same period.

After validating the multiperiod in-sample predictions of insurance demand, the satisfactory predictive performance of the MIDAS model suggests that it can be employed to forecast future insurance demand. Adopting the MIDAS model would likely provide valuable insights for decision-makers in the insurance industry.

## 5. Nowcasting and forecasting

The preceding discussion has established the significant advantage of the MIDAS model in terms of forecast accuracy. In this section, we present evidence that the MIDAS model is capable of nowcasting and forecasting. Using estimates based on the full sample data, Table 6 presents nowcasting and forecasting of China’s insurance demand for the period between 2022Q2 and 2023Q1. The main findings are as follows:

1. Overall, the model’s performance weakens gradually as the horizon increases. This is because the corresponding amount of recent information gradually decreases as the horizon increases, leading to a decrease in the accuracy of predictions. Nevertheless, the forecast values remain within a reasonable range.
2. There is significant volatility in the results of the EPU forecast, which can carry a risk of distortion. The TID values of the CPI and CCI forecast remain relatively stable, and the combination and multivariate predictions outperform the univariate predictions.
3. The TID value has increased to some extent compared to the level of 3.87 in 2022Q1 and is showing a favorable trend, with the expectation of recovering to the level before COVID-19 in 2023Q2. This is mainly due to the normalization of COVID-19, the orderly implementation of various stabilization policies by the Chinese government, the gradual stability of social indicators, the continuous increase in the people’s confidence index in the economy, and the continuous improvement in insurance demand.

**Table 6:** Nowcasting and forecasting.

_Variable_^horizon^		1	2	3	4	5	6	7	8	9	10	11	12
CCI	2022Q2	3.95	3.93	3.92	3.97	3.97	3.96	3.96	3.97	3.94	3.95	3.94	3.94
	2022Q3	--	--	--	3.89	4.04	4.05	4.04	4.05	4.00	3.99	3.98	3.98
	2022Q4	--	--	--	--	--	--	3.84	3.85	4.00	4.03	4.01	4.01
	2023Q1	--	--	--	--	--	--	--	--	--	4.08	4.04	4.01
EPU	2022Q2	3.39	3.46	3.76	3.75	3.71	3.84	3.74	3.82	3.82	3.91	3.82	3.36
	2022Q3	--	--	--	4.35	4.28	4.05	3.91	3.85	3.82	3.96	3.86	3.47
	2022Q4	--	--	--	--	--	--	3.97	3.82	3.87	4.01	3.93	3.63
	2023Q1	--	--	--	--	--	--	--	--	--	4.02	3.97	3.65
CPI	2022Q2	4.00	4.02	4.04	4.01	4.05	4.06	4.04	4.05	4.05	4.03	4.03	3.99
	2022Q3	--	--	--	4.12	4.18	4.21	4.19	4.21	4.20	4.15	4.17	4.11
	2022Q4	--	--	--	--	--	--	4.27	4.30	4.30	4.26	4.28	4.21
	2023Q1	--	--	--	--	--	--	--	--	--	4.34	4.35	4.29
Comb	2022Q2	3.92	3.92	3.95	3.96	3.98	3.99	3.97	3.99	3.97	3.98	3.96	3.90
	2022Q3	--	--	--	4.03	4.12	4.11	4.08	4.09	4.06	4.05	4.04	3.98
	2022Q4	--	--	--	--	--	--	4.02	4.03	4.10	4.12	4.11	4.05
	2023Q1	--	--	--	--	--	--	--	--	--	4.17	4.16	4.09
M(3)	2022Q2	4.00	4.04	4.04	3.98	4.06	4.09	4.03	4.05	4.08	3.94	3.91	3.64
	2022Q3	--	--	--	4.07	4.19	4.25	4.20	4.23	4.26	4.04	4.07	3.89
	2022Q4	--	--	--	--	--	--	4.30	4.34	4.38	4.18	4.29	4.12
	2023Q1	--	--	--	--	--	--	--	--	--	4.26	4.40	4.23
M(2)	2022Q2	4.01	4.00	4.01	4.02	4.02	4.06	4.04	4.06	4.05	4.04	4.03	3.98
	2022Q3	--	--	--	3.99	4.13	4.20	4.20	4.22	4.20	4.16	4.15	4.08
	2022Q4	--	--	--	--	--	--	4.28	4.31	4.30	4.26	4.26	4.19
	2023Q1	--	--	--	--	--	--	--	--	--	4.51	4.51	4.29

‘Comb’ denotes the combined forecast based on BIC-determined weights

‘M(3)’ denotes the multivariate MIDAS function M(3)-AR(1)-MIDAS including CCI, EPU and CPI

‘M(2)’ denotes the multivariate MIDAS function M(2)-AR(1)-MIDAS including CCI and CPI.

## 6. Conclusion and policy implications

In this paper, we try to introduce the MIDAS model into the field of insurance demand forecasting. We select monthly high-frequency data indicators, i.e., CCI, EPU, and CPI, and build a basic MIDAS model by choosing the optimal weighting function and its lag order. In addition to this, we also investigated the impact of different forecast windows and combinations of forecast forms on the forecasting performance. To verify the superiority of the selected MIDAS model, we compare the forecasting performance of the co-frequency AR model and the ARDL model. Finally, the optimal model is used for present and short-term forecasting of future TID values in China.

The following main conclusions are drawn from this study. First, the MIDAS model exhibits higher prediction accuracy compared to the co-frequency AR and ARDL models. Specifically, rolling window and recursive prediction improved the prediction performance, while combined prediction stabilized the prediction results. In addition, the multivariate MIDAS model outperforms the univariate MIDAS model, and the forecasting performance of both models decreases with increasing forecasting horizon, suggesting that the MIDAS model is suitable for short-term forecasting. Secondly, CCI, EPU, and CPI have different explanatory power and predictive effects on TID. Among them, CCI has the best predictive effect and the strongest explanatory power for TID, especially when COVID-19-related uncertainty is included in the estimation interval. The optimal lag order of CCI becomes shorter, and the accuracy of the in-sample prediction improves, suggesting that CCI is the main factor influencing the level of TID during the COVID-19 period and that TID is more sensitive to changes in CCI. Third, from the perspective of nowcasting and short-term forecasting, China’s TID is expected to return to its pre-COVID-19 level in 2023Q1, suggesting that TID will show a positive trend shortly.

In conclusion, the within-sample forecast results show that the mixed-frequency data model has a comparative advantage over the same-frequency data model in the within-sample prediction of China’s insurance demand, and the estimation and prediction results of the model indicate that consumer confidence is an important factor affecting China’s insurance demand in the COVID-19 period; the real-time forecast results suggest that China’s insurance demand will maintain good growth in the post-epidemic era.

The above findings provide several policy insights. First, the Chinese government should refer to consumer confidence indicators in the process of formulating and implementing insurance policies; stabilizing consumer confidence is an important way to secure effective insurance demand. Second, policymakers should combine behavioral indicators with mixed-frequency models, which can provide a better grasp of insurance demand by using the effective information provided by mixed-frequency data. Finally, in the post-epidemic era, China’s insurance market has better prospects, and policymakers should lay out their plans in advance to optimize insurance demand-side and supply-side reforms to safeguard the high-quality development of China’s insurance industry.

This study also has some limitations. For example, this paper only judges whether there is a long-term relationship between CCI, EPU, PPI, and TID from the impulse response results, while it does not explore whether there is a bidirectional causality between the variables and whether there is a problem of covariance; at the same time, this paper does not discuss the limitations of the weighting functions of MIDAS and their impact on the prediction effect. Of course, these contents are not the focus of this paper, and will not affect the correctness of the conclusion.
